# Supplementary material for: Implementation of cervical cancer prevention and screening across five tertiary hospitals in Nepal and its policy implications: A mixed-methods study
Source: PLOS Glob Public Health. 2024 Jan 18;4(1):e0002832. doi: 10.1371/journal.pgph.0002832 (PMC10796028; doi:10.1371/journal.pgph.0002832)
Supplement: S1 Table — (DOCX) [file pgph.0002832.s002.docx]

**S1 Table. Knowledge of cervical cancer among health professionals (N=254)**

| **Characteristics** | **N=254** | |
| --- | --- | --- |
| **Knowledge on risk factors for cervical cancer** | **n** | **%** |
| **Infection with HPV** |  |  |
| Yes | 247 | 97.2 |
| No | 7 | 2.8 |
| **Smoking cigarette** |  |  |
| Yes | 197 | 77.6 |
| No | 57 | 22.4 |
| **Infection with HIV** |  |  |
| Yes | 188 | 74 |
| No | 66 | 26 |
| **Having Multiple deliveries** |  |  |
| Yes | 190 | 74.8 |
| No | 64 | 25.2 |
| **Early age at first delivery** |  |  |
| Yes | 195 | 76.8 |
| No | 59 | 23.2 |
| **Early age at sexual debut** |  |  |
| Yes | 230 | 90.6 |
| No | 24 | 9.4 |
| **Long-term use of oral contraceptives** |  |  |
| Yes | 176 | 69.3 |
| No | 78 | 30.7 |
| **Infection with Chlamydia trachomatis** |  |  |
| Yes | 181 | 71.3 |
| No | 73 | 28.7 |
| **Infection with herpes simplex virus** |  |  |
| Yes | 163 | 64.2 |
| No | 91 | 35.8 |
| **Having multiple sexual partners** |  |  |
| Yes | 242 | 95.3 |
| No | 12 | 4.7 |
| **Over consumption of Alcohol** |  |  |
| Yes | 95 | 37.4 |
| No | 159 | 62.6 |
| **Having a family history of cervical cancer** |  |  |
| Yes | 234 | 92.1 |
| No | 20 | 7.9 |
| **Sign and Symptoms of Cervical Cancer** | | |
| **Foul- smelling vaginal discharge/ smell** |  |  |
| Yes | 239 | 94.1 |
| No | 15 | 5.9 |
| **Bleding after menopause** |  |  |
| Yes | 213 | 83.9 |
| No | 41 | 16.1 |
| **Bleeding after sexual intercourse** |  |  |
| Yes | 232 | 91.3 |
| No | 22 | 8.7 |
| **Abdominal Pain** |  |  |
| Yes | 182 | 71.7 |
| No | 72 | 28.3 |
| **Unexpected Weight Loss** |  |  |
| Yes | 198 | 78 |
| No | 56 | 22 |
| **Having Headache** |  |  |
| Yes | 60 | 23.6 |
| No | 194 | 76.4 |
| **Having a lot of night sweat** |  |  |
| Yes | 85 | 33.5 |
| No | 169 | 66.5 |
| **Cervical Cancer Prevention** | | |
| **Vaccination against HPV** |  |  |
| Yes | 245 | 96.5 |
| No | 9 | 3.5 |
| **Condom Use** |  |  |
| Yes | 225 | 88.6 |
| No | 29 | 11.4 |
| **Avoiding multiple Sex Partner** |  |  |
| Yes | 243 | 95.7 |
| No | 11 | 4.3 |
| **Delaying initiation of sex after 18 years** |  |  |
| Yes | 219 | 86.2 |
| No | 35 | 13.8 |
| **Male circumcision** |  |  |
| Yes | 111 | 43.7 |
| No | 143 | 56.3 |
| **Avoiding prolonged use of oral contraceptive pills** |  |  |
| Yes | 187 | 73.6 |
| No | 67 | 26.4 |
| **Avoid smoking cigarette** |  |  |
| Yes | 212 | 83.5 |
| No | 42 | 16.5 |
| **Avoid multiple delivery/ births** |  |  |
| Yes | 206 | 81.1 |
| No | 48 | 18.9 |
| **Early Screening** |  |  |
| Yes | 251 | 98.8 |
| No | 3 | 1.2 |
| **Total mean score of knowledge (mean, SD)** | 19.83 | 3.5 |
