# Supplementary material for: Implementation of cervical cancer prevention and screening across five tertiary hospitals in Nepal and its policy implications: A mixed-methods study
Source: PLOS Glob Public Health. 2024 Jan 18;4(1):e0002832. doi: 10.1371/journal.pgph.0002832 (PMC10796028; doi:10.1371/journal.pgph.0002832)
Supplement: S2 Table — (DOCX) [file pgph.0002832.s003.docx]

**S2 Table. Attitude about cervical cancer among health professionals**

|  | **Frequency (n)** | **Percentage (%)** |
| --- | --- | --- |
| **I always advise my patients to screen for cervical cancer.** |  |  |
| Strongly disagree | 21 | 8.3 |
| Disagree | 5 | 2 |
| Neutral | 21 | 8.3 |
| Agree | 82 | 32.3 |
| Strongly agree | 125 | 49.2 |
| **I discuss cervical cancer in our staff meeting.** |  |  |
| Strongly disagree | 20 | 7.9 |
| Disagree | 24 | 9.4 |
| Neutral | 65 | 25.6 |
| Agree | 89 | 35 |
| Strongly agree | 56 | 22 |
| **I will be happy to see that my children/sister is immunized against HPV.** |  |  |
| Strongly disagree | 22 | 8.7 |
| Disagree | 1 | 0.4 |
| Neutral | 10 | 3.9 |
| Agree | 66 | 26 |
| Strongly agree | 155 | 61 |
| **Cervical cancer is not a serious health problem, so screening is just a burden.** |  |  |
| Strongly disagree | 173 | 68.1 |
| Disagree | 53 | 20.9 |
| Neutral | 3 | 1.2 |
| Agree | 6 | 2.4 |
| Strongly agree | 19 | 7.5 |
| **Even if we screen and find a woman with precancerous lesion, there is nothing we can do.** |  |  |
| Strongly disagree | 135 | 53.1 |
| Disagree | 81 | 31.9 |
| Neutral | 12 | 4.7 |
| Agree | 12 | 4.7 |
| Strongly agree | 14 | 5.5 |
| **I don't think it is necessary to screen for cervical cancer in our health facility** |  |  |
| Strongly disagree | 169 | 66.5 |
| Disagree | 56 | 22 |
| Neutral | 8 | 3.1 |
| Agree | 5 | 2 |
| Strongly agree | 16 | 6.3 |
| **Government has not shown commitment about cervical cancer so why bother us.** |  |  |
| Strongly disagree | 110 | 43.3 |
| Disagree | 89 | 35 |
| Neutral | 25 | 9.8 |
| Agree | 16 | 6.3 |
| Strongly agree | 14 | 5.5 |
| **I am not interested in cervical cancer prevention because partners/NGOs do their work without developing our capacity to implement cervical cancer control program on our own.** |  |  |
| Strongly disagree | 121 | 47.6 |
| Disagree | 85 | 33.5 |
| Neutral | 24 | 9.4 |
| Agree | 11 | 4.3 |
| Strongly agree | 13 | 5.1 |
| **I will participate in cervical cancer prevention program if I am going to be paid money cash.** |  |  |
| Strongly disagree | 61 | 24 |
| Disagree | 43 | 16.9 |
| Neutral | 57 | 22.4 |
| Agree | 61 | 24 |
| Strongly agree | 32 | 12.6 |
| **I am willing to participate in training for cervical cancer prevention if organized by government of Nepal** |  |  |
| Strongly disagree | 28 | 11 |
| Disagree | 11 | 4.3 |
| Neutral | 8 | 3.1 |
| Agree | 87 | 34.3 |
| Strongly agree | 120 | 47.2 |
| **I am willing to participate in a training for cervical cancer prevention if organized by NGOs** |  |  |
| Strongly disagree | 23 | 9.1 |
| Disagree | 13 | 5.1 |
| Neutral | 10 | 3.9 |
| Agree | 98 | 38.6 |
| Strongly agree | 110 | 43.3 |
| **I am more like to screen women for cervical cancer if I am trained and given equipment & consumables** |  |  |
| Strongly disagree | 28 | 11 |
| Disagree | 14 | 5.5 |
| Neutral | 16 | 6.3 |
| Agree | 89 | 35 |
| Strongly agree | 107 | 42.1 |
| Total mean score attitude (mean, SD) | 47.9 | 8.8 |
