# Supplementary material for: Implementation of cervical cancer prevention and screening across five tertiary hospitals in Nepal and its policy implications: A mixed-methods study
Source: PLOS Glob Public Health. 2024 Jan 18;4(1):e0002832. doi: 10.1371/journal.pgph.0002832 (PMC10796028; doi:10.1371/journal.pgph.0002832)
Supplement: Attachment — Submitted filename: PGPH-D-23-01670_reviewer.pdf [file pgph.0002832.s004.pdf]

PLOS Global Public Health

Implementation of cervical cancer prevention and screening across five tertiary hospitals in Nepal and its policy implications: a mixed-methods study

--Manuscript Draft--

**Manuscript Number:**	PGPH-D-23-01670
**Article Type:**	Research Article
**Full Title:**	Implementation of cervical cancer prevention and screening across five tertiary hospitals in Nepal and its policy implications: a mixed-methods study
**Short Title:**	Implementation of cervical cancer prevention and screening in tertiary hospitals of Nepal
**Corresponding Author:**	Rolina Dhital, MBBS, MHSc, PhD<br>Health Action and Research<br>Kathmandu, NEPAL
**Order of Authors:**	Ganesh Dangal, MBBS, MD, FICS
	Rolina Dhital, MBBS, MHSc, PhD
	Yam Prasad Dwa, MBBS, MD
	Sandesh Poudel, MBBS, MD
	Jitendra Pariyar, MBBS, MD
	Kirtipal Subedi, MBBS, MD
**Keywords:**	Cervical cancer; screening; prevention; implementation; Nepal
**Abstract:**	In Nepal, cervical cancer is the most common cancer among women despite the existing policies. This study intends to assess the implementation of cervical cancer screening through service utilization by women, knowledge and attitude among health professionals, and the perceptions of stakeholders in Nepal. This mixed-methods study was conducted in 2022 across five tertiary hospitals in Kathmandu, Nepal. The quantitative study comprised the health professionals and women attending gynecology outpatient clinics from the selected hospitals. The qualitative study comprised experts on cervical cancer from selected hospitals, civil societies, and the Ministry of Health and Population. The utilization of screening through pap smear among women across five hospitals was 22.2%. Women who were older [adjusted odds ratio (AOR)=1.09, confidence interval (CI): 1.07, 8.19], married (AOR=3.024, CI: 1.12, 8.19), had higher education (AOR=3.024, CI:1.12, 8.42), used oral contraceptive pills (AOR=2.49, CI: 1.36, 4.39), and aware of cervical cancer screening (AOR= 13.28, CI: 6.85, 25.73) were more likely to utilize cervical cancer screening. Among 254 health professionals, higher knowledge was associated with training [Standardized Beta (β)=0.20, CI: 0.44, 2.43)] and having outreach activities in their hospital (β=0.19 CI: 0.89, 9.53) regarding cervical cancer screening. The female health professionals (β=0.16, CI: 0.41, 8.16, P=0.03) and having a cervical cancer screening guideline (β=0.19 CI: 0.89, 9.53, P=0.026) were more likely to have a better attitude for screening. The qualitative findings among 23 stakeholders reflected implementation gaps in policy, supply, service delivery, providers, and community. This study showed low screening among women and specific implementation gaps on cervical cancer prevention and screening across five tertiary hospitals in Kathmandu, Nepal. The findings could help implementing more focused interventions based on the gaps. Intervention studies are also warranted to provide stronger evidence.
**Additional Information:**	
**Question**	**Response**
**Financial Disclosure**	This research do not have specific funding for publication fee.

Powered by Editorial Manager® and ProduXion Manager® from Aries Systems Corporation

Enter a financial disclosure statement that describes the sources of funding for the work included in this submission and the role the funder(s) played. This includes grants and any commercial funding of the work or authors.<br><br>This statement will be typeset if the manuscript is accepted for publication.<br><br>*Please review the submission guidelines and the instructions link below for detailed requirements and guidance.*	
**Competing Interests**<br><br>On behalf of all authors, disclose any competing interests that could be perceived to bias this work.<br><br>This statement will be typeset if the manuscript is accepted for publication.<br><br>*Please review the instructions link below and PLOS Global Public Health's competing interests policy to determine what information must be disclosed at submission.*	None
**Data Availability**<br><br>Before publication, Authors are required to make fully available and without restriction all data underlying their findings. Please see our PLOS Data Policy page for detailed information on this policy.<br><br>A **Data Availability Statement**, detailing where the data can be accessed, is required at first submission. Insert your Data Availability Statement in the box below.	We have uploaded the quantitative data to Figshare with a private link for Editorial and Review Purpose. The link to dataset is https://figshare.com/s/e9247100ffb9900b7099. We shall make the link public upon acceptance.<br>For qualitative data, underlying data is available within the manuscript itself and in supplementary files.

Powered by Editorial Manager® and ProduXion Manager® from Aries Systems Corporation

Please see the data reporting section of our submission guidelines for instructions on what you need to include in your Data Availability Statement.<br><br>This statement will be typeset if the manuscript is accepted for publication.<br><br>*PLOS allows rare exemptions to address legal and ethical concerns. If you have legal or ethical restrictions, please use the box below to detail these in full sentences for the Journal team to consider.*	

Powered by Editorial Manager® and ProduXion Manager® from Aries Systems Corporation

Manuscript

Click here to access/download;Manuscript;Manuscript.docx

Implementation of cervical cancer prevention and screening across five tertiary hospitals in Nepal and its policy implications: a mixed-methods study

Authors

Ganesh Dangal [1,2#], Rolina Dhital[3*#], Yam Prasad Dwa[1, 4$], Sandesh Poudel[1, 5$], Jitendra Pariyar[1,6$], Kirtipal Subedi [1,5$]

[1] Nepal Society of Obstetricians and Gynaecologists, Kathmandu, Nepal

[2]Kathmandu Model Hospital, Kathmandu, Nepal

[3]Health Action and Research, Kathmandu, Nepal

[4]KIST medical college and teaching hospital, Lalitpur, Nepal

[5]Paropakar Maternity and Women's Hospital, Kathmandu, Nepal

[6]Civil Service Hospital of Nepal, Kathmandu, Nepal

*Corresponding author

Email- rolina.dhital@gmail.com

#These authors contributed equally to this work

$These authors also contributed equally to this work

Abstract

In Nepal, cervical cancer is the most common cancer among women despite the existing policies. This study intends to assess the implementation of cervical cancer screening through service utilization by women, knowledge and attitude among health professionals, and the perceptions of stakeholders in Nepal. This mixed-methods study was conducted in 2022 across five tertiary hospitals in Kathmandu, Nepal. The quantitative study comprised the health professionals and women attending gynecology outpatient clinics from the selected hospitals. The qualitative study comprised experts on cervical cancer from selected hospitals, civil societies, and the Ministry of Health and Population. The utilization of screening through pap smear among women across five hospitals was 22.2%. Women who were older [adjusted odds ratio (AOR)=1.09, confidence interval (CI): 1.07, 8.19], married (AOR=3.024, CI: 1.12, 8.19), had higher education (AOR=3.024, CI:1.12, 8.42), used oral contraceptive pills (AOR=2.49, CI: 1.36, 4.39), and aware of cervical cancer screening (AOR= 13.28, CI: 6.85, 25.73) were more likely to utilize cervical cancer screening. Among 254 health professionals, higher knowledge was associated with training [Standardized Beta (β)=0.20, CI: 0.44, 2.43)] and having outreach activities in their hospital (β=0.19 CI: 0.89, 9.53) regarding cervical cancer screening. The female health professionals (β=0.16, CI: 0.41, 8.16, P=0.03) and having a cervical cancer screening guideline (β=0.19 CI: 0.89, 9.53, P=0.026) were more likely to have a better attitude for screening. The qualitative findings among 23 stakeholders reflected implementation gaps in policy, supply, service delivery, providers, and community. This study showed low screening among women and specific implementation gaps on cervical cancer prevention and screening across five tertiary hospitals in

Kathmandu, Nepal. The findings could help implementing more focused interventions based on the gaps. Intervention studies are also warranted to provide stronger evidence.

Introduction

Globally, cervical cancer is the fourth most common cancer in women and continues to be a global public health concern. In 2020, there were an estimated 604,000 new cases of cervical cancer worldwide and about 342,000 women died from the disease [1]. When diagnosed early, cervical cancer is one of the most successfully treatable forms of cancer, as long as it is detected early and managed effectively [2]. The common interventions for cervical cancer screening and prevention include awareness interventions and early medical interventions. The awareness interventions include community-based interventions such as home visits for raising awareness and cultural awareness by health professionals, educational interventions, and media campaigns. The common early medical interventions include pap smears, Human Papilloma Virus (HPV) vaccination, and visual inspection with acetic acid (VIA) [3–6].

The systematic reviews on interventions for cervical cancer screening and prevention are mostly conducted in low and middle-income countries (LMICs) and have indicated positive results globally [4–12]. Studies globally have also identified barriers to cervical cancer screening which include lack of education, low socioeconomic status, lack of knowledge, lack of effective communication, embarrassment, time constraints, and preference for female doctors [13–16]. The studies also highlighted under-representation across different countries, mostly in poorer countries within each region of Africa, Europe, and Asia [4,10,12]. Moreover, the sustainability of the interventions remained inconclusive in the long run.

In Nepal, cervical cancer is the most common cancer among women, accounting for 21.4 percent of all cancers with 80.9% of cervical cancer cases diagnosed in the late stage. The age-standardized yearly incidence of cervical cancer in Nepal is 16.4 per 100,000, making it one of the

countries in South Asia with the highest cervical cancer rates followed by India and Bangladesh [13]. The national guidelines on cervical cancer screening and prevention were introduced in 2010 in Nepal. It suggested screening at least 50 percent of women aged 30–60 years, with recommended screening every five years to reduce cervical cancer mortality by 10% [14].

Most of the studies conducted in Nepal are single-centred hospital-based studies or community-based observational studies [15–20]. The knowledge and utilization of cervical cancer screening remained low in most studies [15–17]. The barriers aligned with the global literature and included poor literacy, lack of awareness, sociocultural aspects, and embarrassment [21–23]. Studies recommended culturally contextual and tailored interventions to improve knowledge and practices [21,24]. However, a comprehensive multicenter study reflecting the users' and providers' perspectives remains scant.

The gap between the high prevalence and lack of preventive strategies for cervical cancer demands an urgent need to scale up preventive actions. Therefore, this study intended to assess the implementation of cervical cancer prevention and screening in five tertiary hospitals in Kathmandu, Nepal. The implementation is assessed through three perspectives: the utilization of prevention and screening services by women, the knowledge and attitude of service providers regarding cervical cancer prevention and screening, and the perceptions of stakeholders working on cervical cancer. The findings could help generate baseline evidence at hospital levels, identify specific gaps and outline the way forward for sustainable interventions.

Methods

Study design

This is a mixed-methods research design that followed a sequential explanatory method where the qualitative approach is intended to provide insights into the quantitative findings.

Study setting and study participants

This study was conducted in five tertiary hospitals in Kathmandu, Nepal representing government, non-government, and teaching hospitals. The hospitals were selected purposively based on the patient flow, feasibility, and accessibility for conducting research. The five hospitals are Paropakar Maternity and Women's Hospital (PMWH), Civil Service Hospital of Nepal (CH), KIST Medical College Teaching Hospital (KIST), Kathmandu Model Hospital (KMH), and Kirtipur Hospital (KH).

For the quantitative study, the population comprised the health professionals and women attending gynecology outpatient clinics of the selected hospitals. The health professionals included the medical doctors and nurses currently working in the obstetrics and gynecology departments of the selected hospitals.

For the qualitative study, the participants comprised the stakeholders representing each of the selected hospitals, representatives from gynecology and oncology societies, and the Ministry of Health and Population (MOHP), and Department of Health Services who are responsible or may have the influence in bringing policy changes related to cervical cancer prevention.

Sample size

Quantitative study

For health professionals, the minimum required sample size was calculated based on a cross-sectional study that was conducted in Uganda [25]. The minimum required sample was calculated to be 241 with a margin error of 5% and a confidence interval of 95%. Considering a 10% non-response rate, the required sample size was calculated to be 268.

For the women attending gynecology OPD, we used the prevalence of 44.9% for the utilization of cervical cancer from a previous study conducted in Nepal [17]. The minimum required sample was calculated to be 379, with a margin of error of 5% and a confidence interval of 95%. Considering that this study will be a multicenter study, we consider each hospital as a cluster and increase the sample size by considering design effects, with a within-hospital-intra-class correlation coefficient of 0.01 for a multicenter study [26]. We considered each hospital as a cluster, and the average number of patients attending gynecology OPD per day was estimated to be 40. Then we calculated the design effect as 1.39, using the formula $Deff = 1+(m-1)\times p$, where m represents the average number of patients attending gynecology OPD each day, and p is the intra-class correlation coefficient. After multiplying the minimum sample size by the design effect, the required number of participants was 526. However, considering a 20% non-response rate, the sample was increased to 657.

Qualitative study

The data was collected until the information reached the point of saturation. In total, data were collected from 23 participants.

Sampling

Quantitative study

For health workers, data was collected through a stratified random sampling method. The health workers were stratified into the doctors' and nurses' groups. From each stratified group, a sampling frame was created where each member was given a unique number. From the sampling frame of participants, 60% of the eligible participants were selected randomly through computer-generated random numbers for nurses and doctors separately for each hospital. The participants from the selected random number were invited to participate in the study. In case of refusal to participate, a new random number was generated for the ones who refused to participate.

For women, data was collected conveniently as it was not feasible to create a sample frame. Women attending the gynecology outpatient clinic who agreed to participate in the study were collected from each hospital. Each hospital collected data from 131 to 132 women from the outpatient clinic during a period of two weeks with an average of 9 to 10 participants enrolled in the study each day in each facility.

Qualitative study

For the qualitative study, the purposive sampling method was performed based on a potential list of participants who had an important role in cervical cancer screening at hospitals and policy levels.

Study variables

The dependent variable for women attending the OPD was their utilization of cervical cancer screening through a pap smear. The independent variables included sociodemographic characteristics and their awareness of cervical cancer, screening of cervical cancer screening, and prevention.

The dependent variables for health professionals included their knowledge of cervical cancer and their attitude towards cervical cancer screening which was assessed by adapting a validated tool used in a study from Uganda [25]. Higher scores indicated better knowledge and attitude. The independent variables included sociodemographic and professional characteristics, training, and the availability of resources for cervical cancer prevention and screening.

Data collection

The data was collected for quantitative and qualitative studies between March 4, 2022 and May 26, 2022.

Quantitative study

The health professionals filled in the self-administered online questionnaire developed in the Kobo toolbox [27]. The questionnaire was sent to the selected doctors and nurses by the research assistants via emails and social media.

The women attending the gynecology outpatient clinic of each hospital were interviewed by the research assistants. The research assistant used a mobile or tablet-based questionnaire using the Kobo toolbox [27]. The research assistants were trained in research ethics and data collection.

Qualitative study

The data collection for the qualitative study adhered to the COREQ guidelines (S1 Table) [28]. A qualified female public health graduate trained in conducting key informant interviews (KII) took the interviews with the key stakeholders. The interviews lasted from 30 minutes to one hour. All interviews were audio recorded with the consent of the participants. The interviewer introduced herself and developed a rapport with the participants. At the end of the interviews, the interviewer summarized the key points with the participants for their feedback. There were no repeat interviews with the same participants.

Data analysis

Quantitative analysis

The quantitative data were analyzed using SPSS version 23. A descriptive analysis of the general characteristics was performed for both the women and health professionals. For women's data, unadjusted and adjusted logistic regression was performed to assess the factors associated with the utilization of cervical screening through a pap smear. For health professionals' data, linear regression was performed to assess the factors associated with knowledge and attitude regarding cervical cancer screening.

Qualitative analysis

Thematic content analysis was performed for qualitative data using Dedoose 8.3.45. The logic model for comprehensive, inter-sectoral cervical cancer prevention based on a scoping review was used as a theoretical framework [29]. The categories were identified by the second author from the coding of the transcripts which were then fitted into five major themes of the logic model framework for cervical cancer prevention. The themes and categories were finalized by all authors. Original anonymous quotes were included to provide more insights.

Ethics statement

This study was approved by Nepal Health Research Council (Proposal ID 34-2022). The study also obtained approval from the institutional review boards of all five study hospitals. Written informed consent was obtained from the participants and the participation was voluntary. The confidentiality of the participants was maintained.

Results

Quantitative results

Table 1 shows the characteristics of the women attending the outpatient clinic of the five hospitals. The mean age of the women was 37.1 (SD 12.2) with the youngest being 18 years and the oldest 79 years of age. The majority of the women were married (81.4%), of Brahmin/Chhetri ethnic groups (50.5%), and were from the Hindu religion (91%). A family history of cancer was reported by 17.8% of women. In total 17.9% were smokers, 22.5% suggested they have used oral contraceptive pills, and 3.8% mentioned they have multiple sexual partners.

216 **Table 1. Characteristics of women.**

217

**Characteristics**	**(N=657)** **n**	**%**
**Age in years (mean SD)**	37.1	12.2
**Marital status**		
Married	535	81.4
Unmarried	122	18.6
**Education**		
Masters	59	9
Undergraduate	176	26.8
Secondary education	143	21.8
Primary education	106	16.1
Literate	74	11.3
Illiterate	99	15.1
**Ethnicity**		
Brahmin/Chhetri	332	50.5

Janajati	228	34.7
Dalit	40	6.1
Others	50	7.6
**Religion**		
Hindu	598	91.0
Buddhist	42	6.4
Others		
**Employment status**		
Employed	222	33.8
Housewife	309	47.0
Agriculture occupation	50	7.6
Others		
**Family history of cancer**		
Yes	117	17.8
No	540	82.2
**Smoking**		

Yes	91	13.9
No	566	86.1
**Oral Contraceptive Use**		
Yes	148	22.5
No	509	77.5
**Multiple sexual partners**		
Yes	25	3.8
No	632	96.2

Table 2 shows the characteristics of the health professionals from the five hospitals. A total of 254 health professionals from the five hospitals participated in the study, of which 171 were nurses and 83 were doctors. The mean age of the participants was 31.9 (SD 7.8) with the youngest being 20 years of age and the oldest 58 years of age. The majority of the respondents were female (87.8%).

Only 37% of the health professionals responded that they had ever been formally trained in conducting screening for cervical cancer. In total, 79.1% responded that they have cervical cancer guidelines for screening in their hospital, 88.6% responded that they have education materials related to cervical cancer screening in their hospital, and 88.6% responded that they have outreach activities for cervical cancer screening in their hospitals.

Table 2. Characteristics of the health professionals.

Characteristics	N=254<br>n	%
**Age (Mean ± S.D) (Maximum, Minimum)**	31.9 ± 7.8	(20, 58)
**Gender**		
Male	31	12.2
Female	223	87.8
**Occupation**		
Nurse	171	67.3
Doctor	83	32.7
**Years of Working (Mean ± S.D)**	7.5 ± 7.0	
**Ever been trained on how to conduct screening for cervical cancer?**		
Yes	94	37
No	160	63
**Does the health facility have any guidelines for cervical cancer screening?**		
Yes	201	79.1
No	53	20.9

**Does this health facility have health education material about cervical cancer?**		
Yes	225	88.6
No	29	11.4
**Do you conduct outreach health education in the community for cervical cancer?**		
Yes	225	88.6
No	29	11.4
* Knowledge score out of a total of 25 scores (Mean ± S.D)	19.8 ± 3.5	
* Attitude score out of a total of 60 scores (Mean ± S.D)	47.9 ±8.8	

*Descriptive data for each question is provided in S2 and S3 Tables

Table 3 demonstrates the unadjusted odds ratios (UOR) and adjusted odds ratios (AOR) in logistic regression models. The model was adjusted for the hospitals, sociodemographic characteristics, risk factors, and if they had heard about cervical cancer screening. In the adjusted model, utilization of cervical cancer screening was found to be associated with an increase in age (AOR =1.13, CI: 1.07, 1.13, P<0.001). Married women were three times more likely to screen for cervical cancer than unmarried women (AOR=3.02, CI: 1.12, 8.19, P=0.03). Women with secondary education were five times more likely (AOR=5.07, CI:1.82-14.09, P=0.002) and women with primary education were three times more likely (AOR=3.02, CI: 1.12, 8.42, P=0.03)

to screen as compared to the illiterate women. Women who have used oral contraceptive pills were also found to be almost two and a half times (AOR=2.49, CI: 1.36, 4.39, P=0.003) more likely to screen for cervical cancer. The women who were aware of cervical cancer screening were 13 times more likely (AOR= 13.28, CI: 6.85, 25.73, P<0.001) to screen as compared to those who had never heard about it.

Table 3. Factors associated with utilization of cervical cancer screening among women.

	UOR	95% CI	AOR	95% CI
**Hospitals**				
CH	1.48	(0.96, 2.29)	1.21	(0.51, 2.83)
KMH	**2.30**	**(1.15, 3.50)***	2.34	(0.97, 5.62)
KH	0.65	(0.39, 1.07)	1.36	(0.54, 3.44)
PMWH	0.89	(0.55, 1.42)	1.33	(0.53, 3.33)
KIST	Ref		Ref	
**Age**	**1.05**	**(1.04,1.07)***	**1.09**	**(1.07, 1.13)***
**Marital status**				
Married	**6.85**	**(2.95, 15.92)***	**3.02**	**(1.12, 8.19)***
Unmarried	Ref			
**Education**				
Masters	1.09	(0.58, 2.06)	2.73	(0.79, 9.39)
Undergraduate	0.68	(0.44, 1.06)	3.04	(0.98, 9.36)
Secondary education	**1.88**	**(1.24, 2.84)**	**5.07**	**(1.82, 14.09)**
Primary education	1.32	(0.82, 2.12)	**3.06**	**(1.12, 8.42)**
Literate	0.79	(0.43, 1.47)	1.61	(0.54, 4.78)
Illiterate	Ref			

**Ethnicity**				
Brahmin/Chhetri	1.29	(0.89, 1.87)	1.07	(0.42, 2.73)
Janajati	1.01	(0.69, 1.49)	1.21	(0.45, 3.27)
Dalit	0.48	(0.18, 1.25)	0.66	(0.15, 2.81)
**Occupation**				
Employed	0.99	(0.67,1.46)	0.75	(0.30, 1.84)
Housewife	1.39	(0.96, 2.01)	0.97	(0.41, 2.28)
Agriculture	**0.28**	**(0.10, 0.80)**	0.35	(0.08, 1.50)
Others	Ref			
**Religion**				
Hindu	1.272	(0.64, 2.52)	4.02	(0.37, 43.49)
Buddhist	1.101	(0.53, 2.29)	5.09	(0.40, 64.60)
Others	Ref			
**Family History of Cancer**				
Yes	**2.59**	**(1.68, 3.99)***	1.35	(0.76, 2.39)
No	Ref		Ref	
**Smoking**				
Yes	0.54	(0.29, 1.01)	0.86	(0.36, 2.08)
No	Ref			
**Contraceptive Use**				

Yes	**1.927**	**(1.28, 2.90)** **	**2.44**	**(1.36, 4.39)** **
No	Ref			
**Multiple sexual partners**				
Yes	1.379	(0.56, 3.37)	1.23	(0.31, 4.84)
No	Ref			
**Ever heard of cervical cancer screening**				
Yes	**9.184**	**(5.44, 15.50)** ***	**13.28**	**(6.85, 25.73)** ***
No	Ref			

***<0.05**

****<0.01**

*****<0.001**

Table 4 demonstrates the adjusted linear regression model that assessed the factors associated with the knowledge and attitude score among health professionals. The sociodemographic characteristics, work experiences related, and training-related variables were adjusted in the model.

Those who were trained in cervical cancer screening (β=0.20, CI: 0.44, 2.43, P=0.005) and those who responded they have outreach activities in their hospital (β=0.16, CI: 0.22, 2.44, P=0.015) were more likely to have higher knowledge about cervical cancer.

The female health professionals (β=0.16, CI: 0.41, 8.16, P=0.03) and those who responded they have a guideline on cervical cancer screening (β=0.19 CI: 0.89, 9.53, P=0.026) were more likely to have a higher attitude for cervical cancer screening. Those who responded they have a treatment facility for cervical cancer (β=-0.17, CI: 7.13, -0.45, P= 0.026), and those who responded they have an outreach activity for cervical cancer screening (β=-0.22, CI: -6.29, -1.62, P=0.001) had a lower score for attitude toward cervical cancer screening.

Table 4. Factors associated with knowledge and attitude among health professionals regarding cervical cancer prevention and screening.

	Knowledge		Attitude	
	**β**	**95% CI**	**β**	**95% CI**
**Age**	-0.26	(-0.23, 0.003)	-0.01	(-0.31 - 0.29)
**Gender**				
Female	-0.01	(-1.63, 1.39)	**0.16**	**(0.41 - 8.16)***
Male	Ref		Ref	
**Occupation**				
Doctor	0.13	(-0.32, 2.28)	0.08	(-1.90, 4.75)
Nurse	Ref			
**Years of experience**	0.20	(-0.03, 0.23)	0.109	(-0.19, 0.46)
**Training on cervical cancer screening**				
Yes	**0.20**	**(0.44, 2.44)****	0.12	(-0.29, 4.79)
No	Ref		Ref	
**Facility guideline on cervical**				

**cancer treatment**				
Yes	0.10	(-0.45, 2.15)	**-0.17**	**(-7.10, -0.45)***
No	Ref		Ref	
**Facility guideline on cervical cancer screening**				
Yes	-0.10	(-2.77, 0.61)	**0.19**	**(0.89, 9.53)***
No	Ref		Ref	
**Health education material in the facility**				
Yes	0.05	(-0.75, 1.67)	-0.01	(-3.39, 2.79)
No	Ref		Ref	
**Community outreach activities in the facility**				
Yes	**0.19**	**(0.22, 2.05)***	**-0.22**	**(-6.29, -1.63)****
No	Ref		Ref	

*<0.05

**<0.01

β = standardized beta coefficient

Qualitative results

Characteristics of the KII participants

We took 23 KII representing the five hospitals, policymakers, and advocates of cervical cancer screening. The details of the characteristics of the participants are provided in Table 5. The majority of the participants were female and represented tertiary hospitals. Four key informants represented the government and were working in the family welfare division (FWD) and national training center (NHTC) of the Department of Health Services, MOHP. The senior practicing gynecologists and obstetricians comprised 43.5%, and the senior nurses comprised 30.4% of the total participants.

Table 5. Characteristics of the key informants.

**Characteristics**	**N=23**<br>**n**
**Gender**	
Female	20
Male	3
**Occupation**	
Practicing Obstetricians and gynecologists	10
Practicing Nurses	7
Government officials from Department of health services, MOHP	4
Non-government Public health and cervical cancer experts	2
**Affiliations**	
Gynecological and oncological societies and foundation	2
Tertiary general hospitals and affiliated medical	14

and nursing schools	
Cancer Specialty Hospitals	3
Department of health services	4

The qualitative findings are structured around five major themes based on the framework of cervical cancer prevention: policy, supply, health system, health service providers, and community (Table 6) [29]. Anonymous original quotes from the responses of the participants are included in the description of the findings to provide more context.

Table 6. Themes based on the framework for cervical cancer prevention.

**Themes**	**Categories**
1. Policy	1.1 Implementation gap<br>1.2 Lack of national HPV vaccination program
2. Procurement and supply	2.1 Tailored implementation<br>2.2 Available resources for trained professionals
3. Health system	3.1 Delivery<br>3.2 Quality
4. Health care providers	4.1 Capacity building<br>4.2 Enabling change

5. Community	5.1 Community engagement<br>5.2 Behavior change communication

1. Policy

Almost all participants acknowledged gaps in the existing policies and implementation for cervical cancer prevention and screening in Nepal.

1.1 Implementation gap

Almost everyone admitted that the implementation gaps have led to low service utilization by women in their hospitals and in general.

"*According to the 2010 national guideline, we should have been way ahead in screening activities till now, but we are still far behind. According to WHO, we should achieve HPV vaccination coverage of 90%, screening of 90%, and treatment of 70% by 2030. However, how will we achieve it in Nepal if the vaccination hasn't been rolled out as part of the national program? The government should take initiatives involving civil societies to work faster and achieve the targets*.'- Gynecologist, and executive, gyne-oncological society.

Some also highlighted the challenges due to the lack of inter-sectoral collaborations leading to implementation gaps. Some opined that private academic institutions are often excluded from government programs despite being an integral part of contributing to cervical cancer elimination in the country.

"There are strategies and policies at the national level. Guidelines and training resources are available. However, private medical colleges are mostly excluded from the national programs.

Many women rely on private teaching hospitals for cervical cancer screening and treatment. We can achieve the targets only if all the sectors are together in the policy implementation."- Gynecologist, private tertiary teaching hospital

1.2 Lack of national HPV vaccination program

Many participants highlighted that delay in vaccination had led to delays in eliminating cervical cancer and achieving the national and global targets. The policymakers informed that the discussions on introducing HPV vaccination as part of the national program are in process. However, they too acknowledged the delay.

"In Nepal, prevalence of cervical cancer is increasing because we haven't been able to do as much we can do. Like primary prevention for cervical cancer is vaccination but vaccination program hasn't started from government's side which should have started by now."- Senior gynecologist, executive, gyne-oncological society

2. Procurement and supply

Many participants suggested while the policy is in place, there should also be a balance between production and supply for proper implementation.

2.1 Tailored implementation

Some suggested that the government should design tailored programs depending on the availability of resources for different health facilities.

"Policymakers should make policies keeping in mind where the updated technology is available and where it is not. Our hospital has a cancer registry and better technologies and facilities than others. One rule that fits all isn't practical for policy implementation. The services provided by

the hospitals with resources and those without are different. Therefore, policy needs to be relevant and contextual to different types of facilities."-Nurse, government tertiary hospital

The health providers also highlighted the lack of availability of the HPV vaccine despite the increasing demand, especially in private hospitals. They pointed out the imbalance between demand and supply.

"The HPV vaccine is in demand here. However, we don't have that facility because it's expensive and not affordable for most people. We cannot purchase the vaccines in bulk and store them for a long time due to their expiry dates, but when a few seek vaccine services, we don't have the vaccines to provide them. The government should include HPV vaccination in its national program to address this imbalance."-Gynecologist, Private hospital

2.2 Available resources for trained professionals

Some also highlighted the need for balance between training coverage and the availability of resources for trained professionals to address the implementation gaps.

"We need to understand that training alone is not a miracle. A trained person needs resources and opportunities to utilize their skills. If doctors receive training on colposcopy, then they should have access to a colposcopy machine where they work. - Senior representative, NHTC

3. Health care system

The participants also provided their insights on improving service delivery and quality of care.

3.1 Delivery

Many suggested that improving the services in one facility alone might not help in health care system. They highlighted the importance of establishing linkages with different facilities for timely referral.

"Frequently VIA screening should be done from the hospital and suspected cases to be referred properly. There should not be any complaint that diagnosis was done but we couldn't get treatment. Referral system should improve so that timely diagnosis and treatment are available."-Gynecologist, Government hospital

3.2 Quality

Many opined that improving the training coverage for health providers could enhance the quality of health services in health facilities. They pointed out that inadequate awareness among health workers remains a challenge.

"The health workers have studied about cervical cancer in their curriculum. However, it doesn't mean they remember everything about cervical cancer screening and prevention forever. So we should provide refresher training regularly to raise awareness among health workers. But the training remains inadequate. The government has provided VIA training so some of them could be more aware. But there are many health workers who haven't received any training and are not sensitized about the importance of cervical cancer prevention and screening. More training coverage should be a priority."- Public health expert, NGO hospital

A few responded that their hospitals are implementing the government policies adequately to improve the quality.

"I can say proudly that our hospital is implementing the policies well. As a head of the department of Gynecology, I have ensured VIA and pap smear for every patient who comes to

our OPD. We also organize outreach activities in the community where we have ensured to perform VIA to promote government's policy. If any woman is detected with cancer, we follow the protocols and refer them for treatment as needed."-Gynecologist, non-government tertiary hospital

4. Providers and healthcare workers

Almost everyone expressed that capacity building of the health workers remains a priority. They acknowledged the challenges of inadequate training coverage and suggested alternate training solutions to enable changes.

4.1 Capacity building

Many reflected on the improvement in health workers' capacity in recent years after the implementation of national policy. They also opined that many health workers are still not trained due to a lack of trainers to expand capacity building.

"The national policy on cervical cancer prevention was introduced only in 2010. There were not many trained professionals back then. But with the collaboration with the government, we developed a pool of trainers. We have continued providing training to nurses and doctors. However, the pool of trained professionals remains inadequate to provide screening services to meet the target of reaching at least 50% of women in Nepal. We are aware of this and working on it."-Gynecologist and national trainer, non-government tertiary hospital

4.2 Enabling change

Some also highlighted that the inadequate trained professionals are due to the turnover of trained professionals in many health facilities and the need to repeat training for recruits. Some

suggested a more efficient way of improving training coverage could be through focused pre-service training when the health professionals are still students.

"We can't tell them to work when they don't know how to work. We can promote pre-service training to the nursing and medical students and incorporate the training package into their clinical rotation in OBGYN. If we could do this, we can add mentoring courses as part of in-service training when they go to work. It could fill the gaps created due to the turnover of trained professionals. Otherwise, the gap in inadequately trained professionals would continue." - Senior representative, NHTC

5. Community

Many also acknowledged that improving the services in the health facilities alone is not enough to strengthen the implementation. They highlighted the importance of establishing community linkages through community engagement and behavior change education strategies.

5.1 Community engagement

Almost everyone expressed that strengthening community engagement would enhance awareness among people. The facilities with community outreach programs also reflected that they could provide better services because of outreach community activities.

"Various outreach awareness activities through virtual or physical presence can improve awareness and demand. I think the outreach activities are helping women utilizing the cervical cancer screening services. However, many women in rural areas are still unaware. Even women living in urban areas like Kathmandu don't prioritize their health. We have been conducting community outreach activities to reach these women and educate them. We are also educating the health workers working in those areas." –Gyne-oncologist, private hospital

5.2 Behavior change communication

Many participants suggested that more efforts in behavior change communication are needed to enhance community awareness. Many opined that public awareness activities are not adequate. *"Information related to cervical cancer in hoarding board similar to smoking prevention would be effective. If we could create media content related to cancer prevention at a larger scale through advertisements, documentaries, dramas, radio programs, etc., then it could help improving awareness. We can learn the lessons of promoting awareness of COVID time. Similar approaches could raise awareness for cervical cancer prevention."*- Nurse, Government tertiary hospital

Discussion

This study showed low utilization of cervical cancer screening and prevention among women attending the outpatient clinics of the selected hospitals. The knowledge among the health professionals was associated with their training and community outreach activities related to cervical cancer prevention and screening in their hospitals. Female health professionals and those having cervical cancer screening guidelines in their hospitals were more likely to have a better attitude toward screening. The qualitative findings complement the quantitative findings and provide further insights into implementation gaps leading to low service utilization among women. The findings highlight the gaps which could encourage more dialogues to execute focused interventions for better implementation.

In this study, the utilization of cervical cancer screening through pap smear among women was only 22% which is much lower than the national target. A systematic review and

meta-analysis on pooled analysis suggested that 17% of women in hospital-based studies and 16% in community-based studies utilized cervical cancer screening in Nepal [30]. The factors associated with screening utilization in this study included age, marital status, education, use of oral contraceptive pills, and awareness about cervical cancer screening. The findings in this study are consistent with previous studies conducted in Nepal and globally. A meta-analysis of the global population from 10 cross-sectional studies showed that a higher level of education improves the odds of women utilizing cervical cancer screening [31]. Studies have shown a similar association of cervical cancer screening utilization with socio-demographic statuses such as age and marital status and awareness of cervical cancer screening [32,33].

In this study, 16% of women were aware of the preventive role of HPV vaccination. However, only 1.5% were vaccinated against HPV. The policy gap due to the lack of a national HPV vaccination program could have contributed to low awareness and HPV vaccine coverage in this study. As of June 2020, 55% of the 194 WHO member countries had already introduced HPV vaccination as part of the national immunization program. However, adequate vaccination coverage is concentrated in high-income countries. Many LMICs like Nepal still haven't introduced the program [34]. As pointed out in the qualitative findings of this study, in Nepal, HPV vaccinations are only available in a few private hospitals where the users need to purchase the vaccine at a higher cost. The key informants from policy levels in this study informed the planning is in place at policy levels and have acknowledged the urgency. However, it is not yet a part of the national immunization program.

The qualitative findings in this study indicated the need for improving hospital-based and community engagement and behaviour-change communication. The interventions on raising

awareness in the hospitals and communities have been proven effective in previous multi-centre studies focusing on hospital-based post-partum family planning in Nepal. At the hospital level, equal access to information for women across all sociodemographic strata through educational videos in the waiting areas outside the outpatient clinic, and group and one-on-one counselling on the importance of cervical cancer screening could be effective [35]. Lessons can be learned from various behaviour change communication interventions for cervical cancer globally and the more recent experiences with the COVID-19 pandemic to enhance the strategies to raise awareness for cervical cancer in Nepal [36–38].

The training of health professionals in this study showed a positive association with better knowledge of cervical cancer screening. However, the training coverage was only 37% in this study. The low coverage of formal training activities among health professionals could have led to low screening among women in this study. A study from Somalia showed that only 22.1% of the participants had cervical cancer training during their pre-service education and 16.8% the in-service training after graduation [39]. In contrast, the study from Turkey indicated that nearly 75% of the health professionals had training as part of their pre-service education, and almost 67% had in-service training on cervical cancer after their graduation [40].

As suggested by the key informants in this study more efforts are needed to promote capacity building for health professionals. An education intervention study from Saudi Arabia for nursing students suggested improved knowledge among nursing students regarding cervical cancer screening after the education intervention [38]. As highlighted by the key informants in this study, incorporating cervical cancer screening in the pre-service curriculum of medical and nursing education and repeated in-service training activities in the hospitals could help improve

the overall knowledge of the health professionals. The locally organized, repeated, hands-on, and simulated training for health service providers in LMICs has been effective in enhancing their skills and confidence with cervical cancer screening, diagnosis, and treatment. Moreover, ongoing sustainable training activities with long-term partnerships of public and private sectors within the health system are necessary to translate health professionals' knowledge into practice [39]. Adapting pre-service and in-service training activities for different maternal and newborn health interventions has proven to be helpful in improving the knowledge and skills of health professionals [41,42]. Similar, strategies could help enable better implementation of cervical cancer prevention and screening.

In this study, the female health professionals and those who responded they have a guideline on cervical cancer screening were more likely to have a better attitude toward cervical cancer screening. Most of the participants in this study were female, and as cervical cancer is a leading cause among women, female professionals could have prioritized the need for screening more as compared to their male counterparts. In contrast, this study also showed those who responded they have a treatment guideline for cervical cancer and community outreach activity for cervical cancer screening in the hospital had a lower attitude toward cervical cancer screening. The hospitals with treatment for cervical cancer could be more focused on the curative aspect rather than the preventive aspects of cervical cancer. Whether the health professionals participating in this study actively participated in the community outreach activities was not explored adequately. The health professionals who were not directly involved in the outreach activities or just involved in treatment may have thought the need to screen has been compensated by the community outreach activities. The qualitative findings in this study have highlighted the importance of community engagement to enhance the implementation. The

majority of the interventions for cervical cancer screening in Nepal and globally are community-based [28,43]. The findings of this study highlight the importance of encouraging health professionals at hospitals to pay equal attention to prevention strategies and also participate in community engagement activities. Moreover, behavioural interventions for health professionals through regular communications and reminders, and training activities could be important steps to be considered by the hospitals to improve the attitude among the health professionals.

Policy implications

The quantitative and qualitative results both indicated implementation gaps to promote cervical cancer prevention and screening utilization. The qualitative study highlighted that the policies are in place but have not been translated into practices as needed. While the key informants from hospital levels highlighted the need for more support and more practical policies from the government, the government representatives acknowledged the challenges of budget allocation, resource limitations, and difficulty in coordination between different levels of government for implementation. Therefore, better coordination and communication are needed between the government and the hospitals to implement targeted interventions at the hospital level alongside community-level interventions. The awareness strategies and service provisions need to be customized based on the services available in different types of health facilities. The hospitals could adopt successful hospital-based health education programs from other reproductive health programs.

The major gap noted in this study also included a lack of adequate training for health professionals affecting their knowledge. Though the pre-service medical and nursing curriculum covers cervical cancer, it is mostly focused on the treatment aspects and the prevention aspects

are not given enough attention. As pointed out by the key informants, a balance between the availability of training and resources for the health professionals to apply their skills must also be considered. The balance between these two aspects must be given more importance through better communication and coordination between the hospitals and different divisions of government that look into training, program implementation, and managing logistics.

Moreover, HPV vaccination coverage remains low attributed to the lack of a vaccination program incorporated into the national immunization program and the high cost of vaccination in private facilities. The key informants who have a crucial role in policymaking have acknowledged the implementation gap and have informed that the dialogues of including it in the national immunization program are in place. However, national HPV vaccination has not yet been introduced and the findings of this reiterate the urgency of promoting it.

Limitations

As a cross-sectional study, the temporal association could not be elicited from this study. Moreover, this study was conducted within the tertiary hospitals of Kathmandu only, and therefore, generalizability to other health facilities across Nepal remains limited. Furthermore, the quantitative study participants for health professionals only included doctors and nurses which limit the insights of other health professionals. Furthermore, over-reporting or under-reporting by the participants cannot be completely ruled out. Despite the limitations, as a multicenter mixed methods study, this study provides deeper insights into the implementation gaps where the qualitative findings support the quantitative findings of low utilization of screening among women and low training coverage within the included hospitals and in general.

As a multicenter study that included major tertiary hospitals with high patient flow, it provides insights into the gaps that could be addressed through future intervention studies.

Conclusions

This study showed low utilization of cancer prevention and screening in the five major tertiary hospitals in Kathmandu, Nepal. The health professionals' knowledge showed significant association with training and having guidelines on screening in their hospitals. However, the training coverage remained low. The attitude for screening needed more effort as well. The qualitative results supported the quantitative results and highlighted the existing policies are not being implemented adequately. The findings could be an important step to initiate dialogues among the policymakers and health professionals to execute the existing policies and work further on addressing the implementation gaps. The findings could pave the way for targeted interventions at tertiary hospital levels. Larger and longitudinal studies are also warranted to provide stronger evidence.

References

1. World Health Organization. Cervical cancer. In: World Health Organization [Internet]. 2022 [cited 11 Oct 2022]. Available: https://www.who.int/health-topics/cervical-cancer

2. World Health Organization. Cervical cancer Fact Sheet. In: World Health Organization [Internet]. 2022 [cited 14 Feb 2022]. Available: https://www.who.int/news-room/fact-sheets/detail/cervical-cancer
3. Majidi A, Majidi S, Salimzadeh S, Khazaee- Pool M, Sadjadi A, Salimzadeh H, et al. Cancer screening awareness and practice in a middle income country; a systematic review from Iran. Asian Pac J Cancer Prev. 2017;18: 3187–3194. doi: 10.22034/APJCP.2017.18.12.3187.
4. Rahman R, Clark MD, Collins Z, Traore F, Dioukhane EM, Thiam H, et al. Cervical cancer screening decentralized policy adaptation: an African rural-context-specific systematic literature review. Glob Health Action. 2019;12: 1587894. doi: 10.1080/16549716.2019.1587894.
5. Saei Ghare Naz M, Kariman N, Ebadi A, Ozgoli G, Ghasemi V, Rashidi Fakari F. Educational interventions for cervical cancer screening behavior of women: a systematic review. Asian Pac J Cancer Prev. 2018;19: 875–884. doi: 10.22034/APJCP.2018.19.4.875.
6. Fokom-Domgue J, Combescure C, Fokom-Defo V, Tebeu PM, Vassilakos P, Kengne AP, et al. Performance of alternative strategies for primary cervical cancer screening in sub-Saharan Africa: systematic review and meta-analysis of diagnostic test accuracy studies. BMJ. 2015;351: h3084. doi: 10.1136/bmj.h3084.
7. Majid U, Kandasamy S, Farrah K, Vanstone M. Women's preferences and experiences of

cervical cancer screening in rural and remote areas: a systematic review and qualitative meta-synthesis. Rural Remote Health. 2019;19: 5190. doi: 10.22605/RRH5190.

8. Mendes D, Bains I, Vanni T, Jit M. Systematic review of model-based cervical screening evaluations. BMC Cancer. 2015;15: 334. doi: 10.1186/s12885-015-1332-8.

9. Mapanga W, Chipato T, Feresu SA. Treatment of cervical cancer in HIV-seropositive women from developing countries: a protocol for a systematic review. Systematic Reviews. 2018;7: 22. doi: 10.1186/s13643-018-0686-9.

10. Jansen EEL, Zielonke N, Gini A, Anttila A, Segnan N, Vokó Z, et al. Effect of organised cervical cancer screening on cervical cancer mortality in Europe: a systematic review. Eur J Cancer. 2020;127: 207–223. doi: 10.1016/j.ejca.2019.12.013.

11. Musa J, Achenbach CJ, O'Dwyer LC, Evans CT, McHugh M, Hou L, et al. Effect of cervical cancer education and provider recommendation for screening on screening rates: a systematic review and meta-analysis. PLoS One. 2017;12: e0183924. doi: 10.1371/journal.pone.0183924.

12. Lu M, Moritz S, Lorenzetti D, Sykes L, Straus S, Quan H. A systematic review of interventions to increase breast and cervical cancer screening uptake among Asian women. BMC Public Health. 2012;12: 413. doi: 10.1186/1471-2458-12-413.

13. Black E, Hyslop F, Richmond R. Barriers and facilitators to uptake of cervical cancer screening among women in Uganda: a systematic review. BMC Women's Health. 2019;19: 108. doi: 10.1186/s12905-019-0809-z.

14. Darj E, Chalise P, Shakya S. Barriers and facilitators to cervical cancer screening in Nepal: a qualitative study. Sex Reprod Healthc. 2019;20: 20–26. doi: 10.1016/j.srhc.2019.02.001.

15. Devarapalli P, Labani S, Nagarjuna N, Panchal P, Asthana S. Barriers affecting uptake of

cervical cancer screening in low and middle income countries: a systematic review. Indian J Cancer. 2018;55: 318–326. doi: 10.4103/ijc.IJC_253_18.

16. Ferdous M, Lee S, Goopy S, Yang H, Rumana N, Abedin T, et al. Barriers to cervical cancer screening faced by immigrant women in Canada: a systematic scoping review. BMC Womens Health. 2018;18: 165. doi: 10.1186/s12905-018-0654-5.

17. Bruni L, Albero G, Serrano B, Mena M, Collado J, Gómez D, et al. Human papillomavirus and related diseases report. Summary Report 2021. ICO/IARC Information Centre on HPV and Cancer (HPV Information Centre); 2021 Oct. Available: https://hpvcentre.net/statistics/reports/NPL.pdf

18. National guideline for cervical cancer screening and prevention in Nepal. Family Health Division, Ministry of health and population.. 2010. Available: https://scholar.google.com/scholar_lookup?hl=en&publication_year=2010&author=Family+Health+Division&title=National+Guideline+for+Cervical+Cancer+Screening+and+Prevention+in+Nepal

19. Thapa N, Maharjan M, Petrini MA, Shah R, Shah S, Maharjan N, et al. Knowledge, attitude, practice and barriers of cervical cancer screening among women living in mid-western rural, Nepal. J Gynecol Oncol. 2018;29: e57. doi: 10.3802/jgo.2018.29.e57.

20. Shrestha AD, Gyawali B, Shrestha A, Shrestha S, Neupane D, Ghimire S, et al. Knowledge, attitude, preventive practices and utilization of cervical cancer screening among women in Nepal: a community-based cross-sectional study. Eur J Cancer Prev. 2022;31: 73–81. doi: 10.1097/CEJ.0000000000000670.

21. Acharya Pandey R, Karmacharya E. Cervical cancer screening behavior and associated factors among women of Ugrachandi Nala, Kavre, Nepal. European Journal of Medical

Research. 2017;22: 32. doi: 10.1186/s40001-017-0274-9.

22. Shakya S, Karmacharya BM, Afset JE, Bofin A, Åsvold BO, Syversen U, et al. Community-based health education has positive influence on the attitude to cervical cancer screening among women in rural Nepal. J Cancer Educ. 2016;31: 547–553. doi: 10.1007/s13187-015-0863-7.

23. Ghimire B, Pathak P. Determinants of uptake of cervical cancer screening among women attending tertiary level hospital. J Nepal Health Res Counc. 2021;18: 649–654. doi: 10.33314/jnhrc.v18i4.2848.

24. Rijal S, Rijal A, Deo SK. Cervical cancer and its screening: a cross-sectional study among married women in Sankhu, Kathmandu. Birat Journal of Health Sciences. 2018;3: 519–523. doi: 10.3126/bjhs.v3i3.22167.

25. Obol JH, Lin S, Obwolo MJ, Harrison R, Richmond R. Knowledge, attitudes, and practice of cervical cancer prevention among health workers in rural health centres of northern Uganda. BMC Cancer. 2021;21: 110. doi: 10.1186/s12885-021-07847-z.

26. Vierron E, Giraudeau B. Design effect in multicenter studies: gain or loss of power? BMC Med Res Methodol. 2009;9: 39. doi: 10.1186/1471-2288-9-39.

27. KoboToolbox (2019) simple, robust and powerful tool for data collection. In: KoboToolbox [Internet]. 2022. [cited 15 Oct 2022]. Available: https://www.kobotoolbox.org/

28. Shrestha AD, Andersen JG, Gyawali B, Shrestha A, Shrestha S, Neupane D, et al. Cervical cancer screening utilization, and associated factors, in Nepal: a systematic review and meta-analysis. Public Health. 2022;210: 16–25. doi: 10.1016/j.puhe.2022.06.007.

29. Damiani G, Basso D, Acampora A, Bianchi CBNA, Silvestrini G, Frisicale EM, et al. The impact of level of education on adherence to breast and cervical cancer screening: evidence

from a systematic review and meta-analysis. Preventive Medicine. 2015;81: 281–289. doi: 10.1016/j.ypmed.2015.09.011.

30. Gizaw AT, El-Khatib Z, Wolancho W, Amdissa D, Bamboro S, Boltena MT, et al. Uptake of cervical cancer screening and its predictors among women of reproductive age in Gomma district, south west Ethiopia: a community-based cross-sectional study. Infectious Agents and Cancer. 2022;17: 43. doi: 10.1186/s13027-022-00455-x.

31. Aynalem BY, Anteneh KT, Enyew MM. Utilization of cervical cancer screening and associated factors among women in Debremarkos town, Amhara region, northwest Ethiopia: community based cross-sectional study. PLoS One. 2020;15: e0231307. doi: 10.1371/journal.pone.0231307.

32. Thapa K, Dhital R, Rajbhandari S, Acharya S, Mishra S, Pokhrel SM, et al. Factors affecting the behavior outcomes on post-partum intrauterine contraceptive device uptake and continuation in Nepal: a qualitative study. BMC Pregnancy and Childbirth. 2019;19: 148. doi: 10.1186/s12884-019-2310-y.

33. Bruni L, Saura-Lázaro A, Montoliu A, Brotons M, Alemany L, Diallo MS, et al. HPV vaccination introduction worldwide and WHO and UNICEF estimates of national HPV immunization coverage 2010–2019. Preventive Medicine. 2021;144: 106399. doi: 10.1016/j.ypmed.2020.106399.

34. Altunkurek ŞZ, Mohamed SH, Şahin E, Yilmaz S. Knowledge and attitudes of healthcare professionals working in a training and research hospital on early diagnosis of cervical cancer (a Somalia example): cross-sectional study. BMC Women’s Health. 2022;22: 228. doi: 10.1186/s12905-022-01808-9.

35. Batkın Revolution S. Investigation of knowledge and attitudes of health workers and

patients in primary health care institutions of Çankaya district towards early diagnosis of cervical cancer. Doctoral dissertation, Ankara Yıldırım Beyazıt University Institute of Health Sciences. 2019.

36. Haider S, Haq N, Riaz S, Nasim A, Saood M, Riffat Y. Nurse's knowledge and awareness regarding cervical cancer and its prevention in different hospitals of Quetta, Pakistan. Research in Pharmacy and Health Sciences. 2018;4: 436–441. doi: 10.32463/rphs.2018.v04i01.06.

37. Dulla D, Daka D, Wakgari N. Knowledge about cervical cancer screening and its practice among female health care workers in southern Ethiopia: a cross-sectional study. Int J Womens Health. 2017;9: 365–372. doi: 10.2147/IJWH.S132202.

38. Mutyaba T, Mmiro FA, Weiderpass E. Knowledge, attitudes and practices on cervical cancer screening among the medical workers of Mulago hospital, Uganda. BMC Medical Education. 2006;6: 13. doi: 10.1186/1472-6920-6-13.

39. Eittah HFA, Aljohani KAS, Aljohani MSE. Enhancing the knowledge of cervical cancer screening among female nursing students: an interventional educational program. Sudan Journal of Medical Sciences. 2020;15: 431–439. doi: 10.18502/sjms.v15i4.8166.

40. Phoolcharoen N, Varon ML, Baker E, Parra S, Carns J, Cherry K, et al. Hands-on training courses for cervical cancer screening, diagnosis, and treatment procedures in low- and middle-income countries. JCO Glob Oncol. 2022;8: e2100214. doi: 10.1200/GO.21.00214.

41. Thapa K, Dhital R, Karki YB, Rajbhandari S, Amatya S, Pande S, et al. Institutionalizing postpartum family planning and postpartum intrauterine device services in Nepal: role of training and mentorship. International Journal of Gynecology & Obstetrics. 2018;143: 43–48. doi: 10.1002/ijgo.12604.

42. Spira C, Dhital R, Jacob S, Dangal G, Gurung G, Prasad Shrestha L, et al. Improving the quality of maternity services in Nepal through accelerated implementation of essential interventions by healthcare professional associations. International Journal of Gynecology & Obstetrics. 2018;143: 379–386. doi: 10.1002/ijgo.12678.

43. Zhang M, Sit JWH, Chan DNS, Akingbade O, Chan CWH. Educational interventions to promote cervical cancer screening among rural populations: a systematic review. International Journal of Environmental Research and Public Health. 2022;19: 6874. doi: 10.3390/ijerph19116874.

Supporting information

S1 Table. COREQ checklist.

S2 Table. Knowledge of cervical cancer among health professionals.

S3 Table. Attitude about cervical cancer among health professionals.

Click here to access/download
Supporting Information
S1 Table COREQ_Checklist.pdf

Click here to access/download
Supporting Information
S2 and S3 Table.docx
