# Supplementary material for: Implementation of cervical cancer prevention and screening across five tertiary hospitals in Nepal and its policy implications: A mixed-methods study
Source: PLOS Glob Public Health. 2024 Jan 18;4(1):e0002832. doi: 10.1371/journal.pgph.0002832 (PMC10796028; doi:10.1371/journal.pgph.0002832)
Supplement: Attachment — Submitted filename: Response to reviewers-cervical cancer (1).docx [file pgph.0002832.s005.docx]

**Response to editor and reviewers**

|  | Editor’s comments | Response |
| --- | --- | --- |
| 1. | Please amend your online Financial Disclosure statement. If you did not receive any funding for this study, please simply state: “The authors received no specific funding for this work.” | We have amended it and mentioned, “The authors received no specific funding for this work.” |
| 2 | Please update your online Competing Interests statement. If you have no competing interests to declare, please state: “The authors have declared that no competing interests exist.” | We have updated the statement as  “The authors have declared that no competing interests exist.” |
|  | Reviewers’ comments |  |
|  | Reviewer #1: |  |
| 1 | Overall, this is a high-quality manuscript that contributes to the knowledge base on cervical cancer prevention and screening in Nepal. It has some minor grammatical errors that need to be corrected before publication | Thank you for your positive response.  We have revised the manuscript and corrected the errors as suggested. |
|  | In Table 2, change “Years of Working” to “Years of work experience”. | Changed in Table 2 |
|  | In Table 3, change “Contraceptive Use” to “Oral contraceptive use”. | Changed in Table 3 |
|  | In line 215, change “oral contraceptive pills” to “oral contraceptives”.  In line 240, change “oral contraceptive pills” to “oral contraceptives”.  In line 251, change “oral contraceptive pills” to “oral contraceptives”. | We have changed “oral contraceptive pills” to “oral contraceptives” throughout the manuscript where applicable. The line numbers have changed due to revisions.  Changed in Line 273 of the revised manuscript, page 14  Changed in Line 305 of the revised manuscript, page 22  Changed Line 525, page 41 of the revised manuscript |
|  | In line 308, change “cervical cancer prevention” to “cervical cancer prevention services”.  In line 314, change “cervical cancer prevention” to “cervical cancer prevention services”.  In line 321, change “cervical cancer prevention” to “cervical cancer prevention services”.  In line 327, change “cervical cancer prevention” to “cervical cancer prevention services”.  In line 333, change “cervical cancer prevention” to “cervical cancer prevention services”.  In line 339, change “cervical cancer prevention” to “cervical cancer prevention services”.  In line 345, change “cervical cancer prevention” to “cervical cancer prevention services”.  In line 351, change “cervical cancer prevention” to “cervical cancer prevention services”.  In line 357, change “cervical cancer prevention” to “cervical cancer prevention services”. | We have now changed “cervical cancer prevention” to “cervical cancer prevention services” throughout the manuscript. The line numbers differ due to revisions.  Lines 78, 81,  Line 126  Line 186  Line 255  Line 358  Line 439  Line 461  Line 506  Line 512  Line 576 |
|  |  |  |
|  | Reviewer #2: |  |
|  | The research is well written and very interesting which focused on current Global health issues. Almost all the work is suitable for publication. Thus, I forwarded some comments for the authors which should be brief for readers to fulfill more standards of publication. I have attached comments on main documents. | Thank you for your encouraging comment and your valuable feedback. We have tried our best to revise the manuscript as per your suggestion. |
|  | 1. Under Methods, there is no Operational definition sub-topic, which is important to make clear how you measured some variables like: knowledge and attitude. | We have now added a sub-topic called “Operational definitions and measurements” under Methods section to provide better clarity for the dependent variables  (Lines 191-216, page 10-12) |
|  | 2. For qualitative study which techniques you used, is it sequential or parallel with quantitative. | The qualitative study was sequential to the quantitative study. We have now clarified it in the Study design under Methods section.  (Lines 89-95, page 6) |
|  | 3. To identify selected nurses or Doctors from the two strata,how did you identified the selected nurse or which coding techniques you used nurses and doctors from five hospitals? make it clear | We have elaborated on how the doctors and nurses were identified and recruited in this study under Methods, Sampling  (Lines 153-168, page 9) |
|  | 4. Knowledge and attitude were measured as linear out come,thus you used linear digression model. . How much level they have? It is not clear ,even from the table of result on knowledge and attitude scale. | We have now provided the details on how the knowledge and attitude was measured and their total score under the Operational definitions and measurements in Methods.  (Lines 191-216, page 10-12) |
|  | 5. Under Discussion You didn't discussed all associated factors. check it .If not you better remove it from table. | We have discussed the significant associated factors for:  Service utilization among women in Discussion Lines 520-527, page 40-41  Factors associated with knowledge among health professionals in Lines 553-564, page 42  Factors associated with attitude among the health professionals in Lines 577-596 |
|  | 6. Also the aim of mixed method is to triangulate the quantitative with KII saying. I haven't seen how you triangulate it inserting in between the quantitative | We have now elaborated on how we employed the sequential explanatory Method under Study design, Lines 89-96. We have included a figure (Fig 1) to provide more clarity on the study flow.  We have also tried to clarify it in the Table 6, as well as in the explanation of the qualitative findings.  We have also discussed key findings in the discussion section based on both quantitative and qualitative findings. |
|  | Reviewer #3 |  |
|  | This research work is commendable for its presentation and meticulousness.  However, I noticed an issue with the alignment of references to their respective sections.  For instance, on line 67, the text discusses cervical cancer incidence in Nepal and cites reference [13],  which is actually a systematic review from Uganda.  Furthermore, on line 454, reference [31] is cited in the context of a global population meta-analysis, but it is, in fact, a community-based cross-sectional study conducted in northwest Ethiopia.  I recommend that the authors carefully review each reference to ensure its alignment with the context in which it is used. | Thank you for your positive response.  Thank you for pointing out the errors in the alignment of the references. We have now re-aligned the references and are now in correct orders.  We have corrected the citation and reference list. The cervical cancer incidence now cites reference [17] which provides Nepal’s data.  We have corrected the reference in lines 523-525 of the revised manuscript with reference [37] which is on the meta-analysis of global data.  We have reviewed the references carefully have revised it accordingly. |
|  | Reviewer #4 |  |
|  | I thank the editor for providing this opportunity to review this article.  After reviewing this article, I have a few suggestions for the authors. | Thank you for our valuable suggestions |
|  | 1. In the introduction section, in lines 53-54, the medical interventions an be grouped as screening vaccination and treatment (the three pillars as per WHO cervical cancer elimination call). In Screening: Include VIA, PAP smear& HPV DNA testing. In treatment include both ablative and excision methods for precancerous and treatment details of cancer. | Thank you for the suggestion. We have revised it as suggested.  Introduction, Lines 53-55, page 4. |
|  | 2. In methods Section, information on the current screening practices in Nepal has to be included. (whether the screening is population –based ?/ who conduct the screening procedure?/which level of health facility?/ what referral mechanism that you have for screen test positives etc) | We have now elaborated on the current screening practices as suggested in the Methods section, under Study settings  Methods, Study Settings, Lines 104-117, page 7. |
|  | 3. Why the tertiary care hospitals are chosen for the study? | We have explained it in the Methods section under Study settings.  Lines 97-103 |
|  | 4. For sample size calculation, study conducted in similar geographical and disease prevalence may be taken | We have cited the study conducted in Nepal to calculate the sample size for service utilization in women.  (Methods, Sample size, Lines 135-137)  However, for health professionals, we adapted the study tools from a study from Uganda and therefore, used it as a reference for calculating sample size.  Moreover, we could not find similar studies that assessed knowledge and attitude among health professionals in Nepal. Thus we chose to refer to the study conducted in LMIC that could be comparable to the context in Nepal |
|  | 5. With the current study results, no recommendations can be made for policy changes. The policy implication paragraph to be removed. | We have now removed the Policy implication paragraph as suggested. |
|  | 6. To rewrite the conclusion (remove details on policy implications) | We have revised the conclusion accordingly removing the policy implications. |
|  | Reviewer #5: I commend the authors for looking at a very important topic. | Thank you for your positive comment. |
|  | Seems there are three aims: assessing barriers among potential patients, among providers and stakeholders.  I would suggest turning this into two manuscripts - the qualitative analysis seems to be a different paper and not sure if all comes together in one coherent manuscript. If the authors decide to keep all aims in this paper, needs significant work. | Thank you for your suggestions. With due respect, this manuscript was intended to be a mixed methods research following the sequential explanatory method.  We have now elaborated on how this research design flows to provide more clarity under Methods section, Study design, Lines 89-95.  We have also added a figure to provide better clarity (Fig 1).  We have tried our best to revise the manuscript and followed your other valuable suggestions carefully. |
|  | Some specific line-by-line feedback for your consideration: | Thank you for providing us such thorough review and line-by-line feedback. |
|  | Abstract |  |
|  | Line 33, if giving number health professionals, would also give number of “women” in line 29. You also give the ‘N” in line 38 (23 stakeholders) | We have now provided N for women as well in Abstract, Line 29 of the revised manuscript |
|  | I’m not sure what it means to have “higher knowledge” (line 33) and the association is with “training?” And “outreach activities” was associated with what outcome (not sure what “regarding cervical cancer screening,” line 35, means)? | We have revised “higher knowledge” to “knowledge score was positively associated with…” to provide better clarity (Abstract, Line 34)  Both training and outreach activities were positively associated with the knowledge score  We have revised the sentence to make it clearer. (Abstract, Lines 33-36) |
|  | Line 35, you mean female as a provider characteristic, versus male? | Yes, we meant female as compared to male. We have revised the sentence to make it clearer. (Abstract, Lines 36-37) |
|  | Line 39-40 – the summary conclusion is about intended patients and stakeholders, does not mention providers, which seems to be another aim? | The stakeholders also included service providers. We have now made it clearer in the revised manuscript.  Abstract, Lines 27-28.  We didn’t elaborate further in the results of the abstract due to word limitation for the abstract. |
|  | Line 42, I would take out the last sentence (“intervention studies are warranted to provide stronger evidence”) – better to end with “… findings could help implement focused interventions…” | We have now removed the sentence as suggested. |
|  | Intro  Line 49-50: common interventions for “screening and prevention” are vaccination (primary prevention) and screening (by cytology, HPV testing and/or VIA) – I am not sure what is meant by “Awareness interventions” or “early medical interventions” | We have now revised the terms to provide better clarity in the Introduction, Lines 49-55, Page 4. |
|  | Line 50-54: I am not sure that “raising awareness” is an evidence-based strategy and there are no references.  I would not call the common screening methods (cytology, HPV and/or VIA) “early medical intervention” – intervention would be perceived as ablation or diagnostic excisional procedure (secondary prevention). | We have added the references for raising awareness which is considered a cost-effective intervention for low resource settings. (Lines 51-53)  We have revised the sentence to make it clearer, that medical intervention can be grouped into screening, vaccination and treatment. (Lines 53-55) |
|  | The first sentence lines 55-57 should be eliminated or re-written – are you saying that screening works? This is well-known and the data are not only in LMICs. Data on screening efficacy initially from high income countries.  Lines 63-64 needs citation  Lines 77-78 – typically “gaps” are when there are evidence based strategies with are NOT, for whatever reason, being utilized. I would guess here that the “gap” is lack of provision of evidence-based interventions to primarily and secondarily vaccinate against and screen for cervical cancer, respectively. The “gap” is not necessarily between prevalence and intervention. | We have revised the sentence and removed LMIC. We meant it is a cost-effective intervention that can also be done in resource constraint settings.  (Lines 56-58)  We have revised the data and sentence and provided the reference accordingly.  (Lines 64-65)  We have now removed the sentence to avoid confusion. |
|  | Methods |  |
|  | Would  Lines 108-111, need to better-explain the sample size calculation – what do you mean it was based on a study in Uganda? The minimal sample to report what?  Line 112, what is “OPD”  Lines 112-123, this is the minimal sample to report what?  Line 148 – what is meant by “awareness”?  Line 191 – how was logistic model built? All variables seem to have been included? | We have revised the sentence to provide more clarity on sample size calculation. We calculated the sample size based on the knowledge score. (Methods, sample size, Lines 130-134)  We have revised to outpatient clinic in the revised manuscript. (Line 235)  We used the proportion of women utilizing cervical cancer screening based on a previous published study in Nepal to calculate the sample size to report minimum required sample size for utilization of cervical cancer screening. (Lines 135-138)  We have revised the term “awareness” to if they “have ever heard of” to provide better clarity under Study Variables, Line 181.  Yes, we have included all the variables in the logistic model. We had chosen the independent variables based on literature review and referring to previous studies. |
|  | Results  Table 3 – adjusted OR are adjusted for all variables in the table? Put in footnote in table. The methods section does not make clear how the model was constructed.  I would not call attention to the p values as you have done – if the CI does not cross one, we know it is a significant OR.  Lines 253-265, what is the statistical test you are doing? This is not clear to me in the methods. How is the “high knowledge of cervical cancer variable Defined? I did not get clarity in methods section.  Starting line 272 through end results – this seems like a different paper  Line 297 – implementation gaps don’t lead to low service utility – the gaps are defined by low usage  Line 301 – this is not he 90-70-90 paradigm, but I suppose it is a quote, so you can’t change, but perhaps use a different quote as this is NOT the 90-70-90 goal. | Footnotes are added for all the adjusted models in Tables 3 and 4.  We have not included p-values in the tables. We have removed the “bold fonts” for the significant values to avoid calling for attention.  Knowledge is treated as a continuous variable. Therefore, the higher scores indicated better knowledge. We removed the term “high knowledge” to avoid confusion.  We have now elaborated how it is measured in the ‘Operational definitions and measurements section’ in the methods section. Lines 188-202.  We have also mentioned that we conducted linear regression in Data analysis, Lines 248-249  We have now elaborated on how we employed the sequential explanatory Method under Study design, Lines 89-96. We have included a figure (Fig 1) to provide more clarity on the study flow.  We have also tried to clarify it in the Table 6, as well as in the explanation of the qualitative findings.  The themes were identified based on the responses from the stakeholder. As outlined by the stakeholders, it could go both ways. In this study, it highlights additional efforts have not been done in terms of implementation due which many women are not aware of the usage of the service utilization.  We have now removed the quote to avoid confusion. |
|  | Discussion  Line 444-445 – last sentence of this paragraph is repetitive , rewrite or eliminate.  Line 534 – this is not how we typically define a “gap”  542 – I think this is the first time we are hearing about vaccination, this should come much earlier  Line 569-570 – take out last sentence | We have removed the sentence as suggested  We have removed to the entire “Policy implications” section based on Reviewer #4’s suggestions  This line has already been removed. However, we have discussed about HPV vaccination in the Discussion (Lines 529-542)  We have removed the last sentence from conclusion as suggested. |
|  | Reviewer #6: The study is important because it addresses a potentially lethal pathology, cervical cancer, which can be cured in the early stages in practically 100% of cases.  One minor detail: the “other” data was missing in Table 1 - characteristics of women, in the topic religion and employment status.  The proposal to carry out a mixed study is interesting to exemplify which approaches can be more effective in health teams that deal directly with this, raising awareness of the need to take advantage of any opportunity for diagnosis as early as possible or even qualifying vaccination, in addition to training specific for prevention, diagnosis and treatment related to the topic, integrating the public and private, overcoming the dichotomy between preventive and curative action, improving cure rates. Greater community involvement in conjunction with what has already been described leads to the implementation of improvements in health policies. | Thank you for your encouraging comments.  Thank you for pointing this error. We have added the values for others.  Thank you for your comment and we completely agree with you. |
